# Supplementary material for: Behaviour change interventions for the management of Raynaud’s phenomenon: a systematic review protocol
Source: BMJ Open. 2017 Aug 4;7(8):e017039. doi: 10.1136/bmjopen-2017-017039 (PMC5724227; doi:10.1136/bmjopen-2017-017039)
Supplement: Supplementary file 1 [file bmjopen-2017-017039supp001.pdf]

Supplementary Table 1: PRISMA-P Checklist with manuscript page number reference

Section and topic	Page	Item No	Checklist item
**ADMINISTRATIVE INFORMATION**			
Title:			
Identification	1,2,3	1a	Identify the report as a protocol of a systematic review
Update	NA	1b	If the protocol is for an update of a previous systematic review, identify as such
Registration	2,4	2	If registered, provide the name of the registry (such as PROSPERO) and registration number
Authors:			
Contact	1	3a	Provide name, institutional affiliation, e-mail address of all protocol authors; provide physical mailing address of corresponding author
Contributions	9	3b	Describe contributions of protocol authors and identify the guarantor of the review
Amendments	NA	4	If the protocol represents an amendment of a previously completed or published protocol, identify as such and list changes; otherwise, state plan for documenting important protocol amendments
Support:			
Sources	9	5a	Indicate sources of financial or other support for the review
Sponsor	9	5b	Provide name for the review funder and/or sponsor
Role of sponsor or funder	9	5c	Describe roles of funder(s), sponsor(s), and/or institution(s), if any, in developing the protocol
**INTRODUCTION**			
Rationale	3	6	Describe the rationale for the review in the context of what is already known
Objectives	3	7	Provide an explicit statement of the question(s) the review will address with reference to participants, interventions, comparators, and outcomes (PICO)
**METHODS**			
Eligibility criteria	4	8	Specify the study characteristics (such as PICO, study design, setting, time frame) and report characteristics (such as years considered, language, publication status) to be used as criteria for eligibility for the review
Information sources	5 Online sup 2	9	Describe all intended information sources (such as electronic databases, contact with study authors, trial registers or other grey literature sources) with planned dates of coverage
Search strategy	5 Online sup 2	10	Present draft of search strategy to be used for at least one electronic database, including planned limits, such that it could be repeated
Study records:			
Data management	5	11a	Describe the mechanism(s) that will be used to manage records and data throughout the review
Selection process	5	11b	State the process that will be used for selecting studies (such as two independent reviewers) through each phase of the review (that is, screening, eligibility and inclusion in meta-analysis)
Data collection process	5	11c	Describe planned method of extracting data from reports (such as piloting forms, done independently, in duplicate), any processes for obtaining and confirming data from investigators

Data items	4, 5	12	List and define all variables for which data will be sought (such as PICO items, funding sources), any pre-planned data assumptions and simplifications
Outcomes and prioritization	4, 5	13	List and define all outcomes for which data will be sought, including prioritization of main and additional outcomes, with rationale
Risk of bias in individual studies	6	14	Describe anticipated methods for assessing risk of bias of individual studies, including whether this will be done at the outcome or study level, or both; state how this information will be used in data synthesis
Data synthesis	7	15a	Describe criteria under which study data will be quantitatively synthesised
	7	15b	If data are appropriate for quantitative synthesis, describe planned summary measures, methods of handling data and methods of combining data from studies, including any planned exploration of consistency (such as I^2, Kendall's τ)
	7, 8	15c	Describe any proposed additional analyses (such as sensitivity or subgroup analyses, meta-regression)
	7	15d	If quantitative synthesis is not appropriate, describe the type of summary planned
Meta-bias(es)	NA	16	Specify any planned assessment of meta-bias(es) (such as publication bias across studies, selective reporting within studies)
Confidence in cumulative evidence	6	17	Describe how the strength of the body of evidence will be assessed (such as GRADE)

Supplementary Table 2 & 3: Search Strategy

Search engines used: Medline, Embase, PsychINFO, Cochrane (see table 1 and 2 for search terms and strings for respective search engines).

Table 2: Medline (PubMed), PsychINFO, Cochrane Search terms

Number	Strategy
#1	"raynaud's disease" OR Raynaud* OR "Raynaud's phenomenon"
#2	cognitive therap* OR behavior therap* OR Biofeedback OR Psychotherap* OR "Clinical psychology" OR self-management OR non-pharmacological OR "Cognitive Behavioral Therapy" OR "Cold avoidance" OR "behavior* medicine" OR "psychological intervention*" OR "psychoeducation" OR "education"
#3	#1 AND #2

Note. Searching for above terms within 'title and article' and only clinical trials; * = truncation to find plurals, alternative spellings and related concepts.

Table 3: Embase Search Strategy

Number	Strategy
#1	'raynaud phenomenon'
#2	'cognitive therapy' OR 'behavior therapy' OR 'feedback system' OR psychotherapy OR 'clinical psychology' OR 'self care' OR non-pharmacological OR 'cognitive therapy' OR 'cold avoidance' OR 'behavioral medicine' OR 'psychological intervention' OR 'psychoeducation' OR 'education'
#3	#1 AND #2

Note. Searching for above terms within 'title and article' and only clinical trials; * = truncation to find plurals, alternative spellings and related concepts.

Supplementary Table 4: Data extraction

Systematic review of behaviour change interventions for Raynaud's Phenomenon: Data Extraction Form

**Citation** (include live link)		JD
		JP
**Retrieval information (date/location)**		CE

Eligibility criteria

**Adults with Raynauds (primary or secondary)**	
**Randomised controlled trial**	
**At least one active behaviour change intervention and control**	

Study Details

**Patient population**	**Primary RP (n)**	**Systemic sclerosis (n)**	**Other secondary RP (describe in box below)**
**Diagnostic criteria used**			
**Year of publication**			
**Study design**			
**Format of Intervention**			
**Study setting / country**			
**Sample characteristics (incl. size & subgroup)**			
**Gender**			
**Age**			
**Ethnicity**			
**Active treatment Interventions**			
**Pre-defined behaviour change model/theory**	Yes / No	Please state:	
**Control intervention**			
**Duration of study**			
**Primary end-points**			
**Secondary end-points**			
**No. treatment sessions**			
**Level of therapist training**			
**Integrity of the intervention checked?**			
**Primary Outcomes:**			
**Frequency of attacks**			
**Duration of attacks**			
**Severity of attacks**			
**Pain**			

**Patient assessment of disability**	
**Adverse events**	
**Withdrawal**	
**Secondary Outcomes:**	
**Physician global assessment of severity**	
**Patient global assessment of disability**	
**Change in digital ulceration**	
**Treatment preference**	
**General improvement**	
**Anxiety**	
**Depression**	

**Comments**

Supplementary table 5: risk of bias assessment

Study Validity Domains	Assessment	Comments
1. Sequence Generation: Was the allocation sequence adequately controlled?	Yes ☐<br>No ☐<br>Unclear ☐	
2. Allocation concealment: Was the sequence generation adequately concealed before group assignments?	Yes ☐<br>No ☐<br>Unclear ☐	
3. Blinding of participants and personnel: Was knowledge of the allocated interventions adequately hidden from the participants and personnel after the participants were assigned to respective groups?	Yes ☐<br>No ☐<br>Unclear ☐	
4. Blinding of outcome assessors: Was knowledge of the allocated interventions adequately hidden from the outcome assessors after participants were assigned to respective groups	Yes ☐<br>No ☐<br>Unclear ☐	
5. Incomplete outcome data: Were incomplete outcome data adequately addressed?	Yes ☐<br>No ☐<br>Unclear ☐	
6. Selective outcome reporting: Are reports of the study free of suggestion of selective outcome reporting?	Yes ☐<br>No ☐<br>Unclear ☐	
7. Other sources of bias: Was the study apparently free of other problems that could put it at a risk of bias?	Yes ☐<br>No ☐<br>Unclear ☐	
