# Supplementary material for: Behaviour change interventions for the management of Raynaud’s phenomenon: a systematic review protocol
Source: BMJ Open. 2017 Aug 4;7(8):e017039. doi: 10.1136/bmjopen-2017-017039 (PMC5724227; doi:10.1136/bmjopen-2017-017039)
Supplement: Author's manuscript [file bmjopen-2017-017039.draft_revisions.pdf]

BMJ Open is committed to open peer review. As part of this commitment we make the peer review history of every article we publish publicly available.

When an article is published we post the peer reviewers' comments and the authors' responses online. We also post the versions of the paper that were used during peer review. These are the versions that the peer review comments apply to.

The versions of the paper that follow are the versions that were submitted during the peer review process. They are not the versions of record or the final published versions. They should not be cited or distributed as the published version of this manuscript.

BMJ Open is an open access journal and the full, final, typeset and author-corrected version of record of the manuscript is available on our site with no access controls, subscription charges or pay-per-view fees (http://bmjopen.bmj.com).

If you have any questions on BMJ Open's open peer review process please email editorial.bmjopen@bmj.com

Behaviour change interventions for the management of Raynaud's phenomenon: a systematic review protocol

Journal:	*BMJ Open*
Manuscript ID	bmjopen-2017-017039
Article Type:	Protocol
Date Submitted by the Author:	28-Mar-2017
Complete List of Authors:	Daniels, Jo; University of Bath, Dept. Psychology<br>Pauling, John; University of Bath , Dept. Pharmacy and Pharmacology ; Royal National Hospital For Rheumatic Diseases NHS Foundation Trust, Rheumatology Department<br>Eccleston, Christopher; University of Bath, Centre for Pain Research
**Primary Subject Heading**:	Rheumatology
Secondary Subject Heading:	Rheumatology
Keywords:	RAYNAUD'S PHENOMENON, behaviour change, Systematic review

SCHOLARONE™
Manuscripts

Behaviour change interventions for the management of Raynaud's phenomenon: a systematic review protocol

Daniels, J.[1] Pauling, J.D.[2,3] & Eccleston, C.[4]

Word count (excluding references and abstract): 2037

[1] Department of Psychology, The University of Bath, Claverton Down, Bath, BA2 7AY, United Kingdom.

[2] Department of Rheumatology, Royal National Hospital for Rheumatic Diseases, Upper Borough Walls, Bath BA1 1RL

[3]Department of Pharmacy and Pharmacology, The University of Bath, Claverton Down, Bath, BA2 7AY, United Kingdom.

[4] Centre for Pain Research, Department for Health, The University of Bath, Bath, BA2 7AY, United Kingdom.

*Requests for reprints should be addressed to Dr Jo Daniels, University of Bath, Department of Psychology, Claverton Down, Bath BA2 7AY, UK, t: 01225385506 e: j.daniels@bath.ac.uk

For peer review only - http://bmjopen.bmj.com/site/about/guidelines.xhtml

ABSTRACT

Introduction: Raynaud's Phenomenon (RP) describes excessive peripheral vasospasm to cold exposure and/or emotional stress. RP episodes are associated with digital colour changes, pain and reduced quality of life. Pharmacological interventions are of low to moderate efficacy and often result in adverse effects such as facial flushing and headaches. Recommended lifestyle and behavioural interventions have not been evaluated. The objectives of the proposed systematic review are to assess the comparative safety and efficacy of behaviour change interventions for RP and identify what we can learn to inform future interventions.

Methods and analysis: Studies eligible for inclusion include randomised controlled trials (RCTs) testing a behaviour change interventions with control comparator. A comprehensive search strategy will include peer-review and grey literature up until 30th April 2017. Search databases will include Medline, Embase, PsychINFO and Cochrane. Initial sifting, eligibility, data extraction, risk of bias and quality assessment will be subject to review by two independent reviewers with a third reviewer resolving discrepancies. Risk of bias assessment will be performed using Cochrane risk of bias assessment tool with quality of evidence assessed using GRADE. A meta-analysis will be performed if there are sufficient data. Two subgroup analyses are planned: primary vs. secondary RP outcomes; comparison of theoretically-informed interventions with pragmatic interventions.

Ethics and dissemination: This review does not require ethical approval as it will summarise published studies with non-identifiable data. This protocol complies with 'Preferred Reporting Items for Systematic Review and Meta-Analysis Protocols (PRISMA-P) guidelines. Findings will be disseminated in peer-reviewed articles and reported according to PRISMA. This review will make a significant contribution to the management of RP where no review of behaviour-change interventions currently exist. The synopsis and protocol for the proposed systematic review is registered in the International Prospective Register of Systematic Reviews (registration number CRDXXX).

For peer review only - http://bmjopen.bmj.com/site/about/guidelines.xhtml

STRENGTHS AND LIMITATIONS

- This study will offer the first review of behaviour-change interventions for the treatment of Raynaud 's phenomenon, making a significant contribution to our current understanding of treatment options and interventions for this common medical complaint.
- Risk of bias assessment will be performed using Cochrane risk of bias assessment tool with quality of evidence assessed using GRADE. We will also assess for quality of each psychotherapeutic intervention, further comparing theoretically-underpinned and pragmatic interventions.
- We will restrict our review to randomised controlled trials only, which may exclude studies of potential interest. However, we aim to evaluate efficacy of high quality interventions and use the outcomes of this review to inform future high quality interventions for Raynaud's Phenomenon, therefore we believe this exclusion is appropriate. We intend to discuss noteworthy non-randomised trials or observational studies, however this will not be part of our specified search.

Key words: Raynaud's Phenomenon, behaviour change, systematic review

For peer review only

For peer review only - http://bmjopen.bmj.com/site/about/guidelines.xhtml

INTRODUCTION

Peripheral vasoconstriction of thermoregulatory pre-capillary arterioles and arteriovenous anastomoses is a normal physiological response to cold exposure designed to preserve normal core temperature.[1] Raynaud's Phenomenon (RP) describes excessive peripheral vasospasm to cold exposure and/or emotional stress. Attacks of RP are associated with characteristic digital colour changes (reflecting blood oxygenation and tissue perfusion). Tissue ischaemia (and subsequent reperfusion) during attacks of RP results in pain and paraesthesia causing distress, loss of hand function and reduced quality of life. RP is common, affecting approximately 10% of people.[2] The majority of RP sufferers have a functional vasospastic disorder that, whilst intrusive, is otherwise benign in nature (termed primary RP). Digital perfusion is generally normal in between attacks. In other circumstances, symptoms of RP occur as a result of disturbed digital tissue perfusion related to separate underlying pathology (termed secondary RP). Important causes of secondary RP are the autoimmune rheumatic diseases such as systemic sclerosis (SSc). In SSc, endothelial damage leads to an obliterative microangiopathy, characterised by structural changes to the vessel wall and near permanent tissue ischaemia. SSc, despite being rare with an estimated prevalence of 250/million,[3] is often used as the focus of RP research. Cold exposure is the most frequent precipitating factor, although emotional stress may account for up to 1/3 of RP attacks.[4]

Behavioural approaches to RP management include lifestyle and habit reversal interventions such as avoiding cold exposure, conserving heat loss, smoking cessation, increasing exercise, and reducing stress levels.[5] It is recognised that adherence with interventions of this nature can be poor with estimates of 30-50% of patients demonstrating poor compliance, regardless of condition, expected outcome, or setting.[6] Pharmacological interventions to induce vasodilation and prevent excessive vasoconstriction of the digital microvasculature also have a role in RP management but the efficacy of such treatments is modest in terms of frequency and severity of RP attacks.[7] Furthermore, pharmacological intervention to promote vasodilation often results in adverse effects such as facial flushing, headaches, fluid retention, dizziness, and palpitations.[8] Despite the perceived importance of non-pharmacological interventions, comparative efficacy, adoption and compliance with lifestyle interventions has not been fully evaluated.[8] A number of behavioural interventions have been tested for RP but the comparative efficacy of a range of interventions within different disease populations (primary and secondary RP) has not previously been the focus of a systematic review. The specific objectives of the proposed systematic review are to assess the comparative safety and efficacy of a range of behavioural interventions for the management and treatment of symptoms

For peer review only - http://bmjopen.bmj.com/site/about/guidelines.xhtml

associated with primary and secondary RP. The secondary objective is to identify what we can learn from the studies reviewed to inform future behaviour change interventions for RP.

METHODS AND ANALYSIS

Reporting of protocol and review registration.

This protocol follows PRIMSA-P guidelines[9] for the reporting of a systematic review protocol (see Online Supplementary table 1). The synopsis and protocol for this systematic review is registered in the International Prospective Register of Systematic Reviews (http://www.crd.york.ac.uk/PROSPERO)[12] registration number CRD – tbc. Reference was also made to the generic Cochrane protocol for pharmacological interventions for the treatment of RP.[8]

Study Selection

Randomised Controlled Trials (RCTs) testing one or more active treatment interventions with a control comparator arm will be included. Both individual and cluster randomisation will be included providing cluster sites meet other inclusion criteria. Non-randomised controlled trials and other intervention study designs will not be included. Blinding in non-pharmacological interventions is not always possible or relevant. Non-blinded studies will be included.

Participants

Adults (18 years or older) with a diagnosis of Raynaud's Phenomenon (primary or secondary). Mixed samples studies will be included. The search will be limited to studies with humans

Interventions

We define behaviour change interventions as interventions which target symptomatic relief of RP through directed or advised change in patient determined behaviour. We are particularly interested in studies consistent with NICE guidelines for RP,[5] however we purposefully include all interventions which are designed to change behaviour to improve symptoms. Behaviour change interventions are described variably and may use the following adjectives, all of which will be used in the search strategy: behavio(u)ral therapy, cognitive therapy, education, psychoeducation, biofeedback, clinical psychology, psychotherapy, self-management, cognitive behavio(u)r therapy, and behavioural medicine.

Outcome measures

There is no consensus over the domains of measurement or measurement technology.[10] We adopt Pope et al.'s,[8] approach and identify primary outcomes domains as: frequency (average daily or

For peer review only - http://bmjopen.bmj.com/site/about/guidelines.xhtml

weekly attack frequency), duration (average duration in minutes), severity/impact of RP episodes; pain (intensity of pain during attack); and patient global assessment of disability. These outcomes are measured by the Raynauds Condition Score (RCS),[11] or equivalent (e.g. Raynaud's severity scale patient symptom monitoring which preceded the RCS). Adverse events (hospitalization/death) are also included within our primary outcomes. Withdrawals from study will also be noted. Secondary outcomes domains are: daily functioning; RCS composite score; physician global assessment; change in digital ulceration (positive/negative); treatment preference; general improvement (self-reported overall improvement).

Search Strategy

A comprehensive search strategy has been created, which includes searching of grey-literature. Four sources of peer-review databases will be used: Medline, Embase, PsychINFO and Cochrane. The search will have no language or publication date restrictions, seeking to translate where necessary. Studies must be fully published at the time of search; early view online publications will be included. A draft search strategy is included in the online supplementary table 2 & 3. Endnote and Covidence software (covidence.org) will be used for data management.

Selection of Studies

All studies generated by the search will be screened by two review authors for eligibility. Full texts will be retrieved for those which meet eligibility, which will then be rated independently by two review authors using a pre-specified data extraction form (online supplementary tables 4 & 5). Bibliographies of included studies will also be searched for inclusion of relevant studies, which will then be subject to independent review of eligibility. Discrepancies will be reviewed, with resolution through discussion and consultation of a third author reviewer. Level of agreement will be calculated.

Data collection process

All eligible studies will be subject to independent data extraction by two review authors. A data extraction form has been developed and piloted to extract the following: full citation including author and year of publication; retrieval information(date/database); patient population; diagnostic criteria used for RP diagnosis; study design; intervention(s); study setting/county (including language); format of intervention (e.g. group/individual/internet); sample size and description; age/gender/ethnicity/ intervention(s); duration of study; number of treatment sessions; primary endpoints; secondary outpoints; frequency, duration and severity of RP attacks; outcome/results; comments, adverse events.

For peer review only - http://bmjopen.bmj.com/site/about/guidelines.xhtml

We will also collect information pertaining to the quality of psychotherapeutic interventions: (a) reference to a theoretical model (b) level of therapist training (c) whether the integrity of the intervention was checked. These criteria are drawn from an authoritative review of empirically supported psychotherapies .[12] We will attempt to retrieve missing data with study authors.

The data extraction form will feature a brief eligibility check box with core inclusion criteria: adults with RP; behaviour change intervention with at least one comparator; RCT; RCS or equivalent. All criteria need to be present in order to demonstrate eligibility.

Risk of Bias

Two review authors will independently assess risk of bias on an outcome and study level using the Cochrane risk of bias assessment tool. Unresolved discrepancies will be reviewed by the third review author. We will assess risk of bias according to the following dimensions: random sequence generation (adequate description and method of participant allocation in accordance with standard randomisation); allocation concealment (adequate concealment of group assignment to prevent selection bias); blinding of participants and personnel (adequacy of measures taken to prevent performance bias and conceal group assignment); blinding outcome assessors (adequacy of measures taken to prevent detection bias and conceal group assignment to outcome assessors); incomplete data (adequacy of the management of missing data and potential implications for bias); selective outcome reporting (reporting bias relating to the consistency between pre-specified and reported outcomes); other sources of bias (other concerns not covered elsewhere but may lead to a risk of bias). All eligible studies will be rated as high low, or unclear (risk of bias), on each of these dimensions, culminating in an overall risk of bias (high/low/unclear) in accordance with the Cochrane handbook for systematic reviews of interventions.[13] Funnel plots will be used to assess bias and will be stratified based on bias assessment, if considered useful and appropriate (>10 studies).

Quality of evidence

We will assess the quality of the evidence, and so our confidence in any estimates of effect using GRADE: (in)consistency of effect, imprecision, indirectness and publication bias. [14] If possible we will construct a GRADE summary of findings table, using the seven primary outcomes. When possible we will express dichotomous outcomes (e.g. no less than mild pain defined as <30 on a 100 point scale,[15] or 50% reduction in the number of episodes, as NNT, or minimal importance clinical differences.[16]

For peer review only

For peer review only - http://bmjopen.bmj.com/site/about/guidelines.xhtml

ANALYSIS

We will perform a meta-analysis if there are sufficient data, and it is clinically sensible.

Effect sizes will be calculated at post-test between therapies for each comparison with control (using hedges g) by subtracting average score of active arm from average of control arm and dividing by pooled standard deviation. We will also calculate mean/median differences from baseline to post treatment for each invention and mean differences of change in measures associated with primary outcomes (where appropriate).

The review outcomes are likely to be reported in both dichotomous and continuous data. For dichotomous data we will report the results as summary of risk ratios and confidence interval (CI) levels of 95%, pooling those data with identical outcomes and interventions. For continuous data, standardised mean difference and 95% CI will be used due to likelihood of discrepant scales used in the study. The Cochrane handbook will be used for translation of outcomes of scales.

Heterogeneity will be assessed on the basis of study design, participants, interventions and outcomes and will be assessed using forest plots (using 10% level). Higgins I^2 statistic, X^2, and visual inspection of forest plots will be used to assess homogeneity with thresholds of 50-75% for moderate heterogeneity and 75% for significant heterogeneity. We anticipate the use of random-effects meta-analysis due to the likely heterogeneity of participant and outcomes data however fixed models will be used where appropriate.

In the absence of a meta-analysis, a narrative (descriptive) analysis of primary and secondary outcomes will be provide and will include: 1. Aggregated data on mean daily frequency/severity/duration of episodes, patient global assessment of disability and changes on RCS scores. 2. Analysis of reported design or intervention flaws in behaviour change interventions 3. Analysis of reported considerations as regards of future behaviour change interventions.

Due to the lack of reliable endpoints in RP treatment trials, we will specify minimally important differences (MIDs) in measures to allow clinically meaningful interpretation of change on standardised measures: 15-point difference on RCS, 20% difference on VAS and likert scales (based previous estimates in RP [10]) use of reliable change index (RCI)[17] for other standardised measures.

Planned sub-group analysis.

For peer review only - http://bmjopen.bmj.com/site/about/guidelines.xhtml

Two subgroup analyses are planned based on specific clinical hypotheses. First, we will analyse outcomes by diagnosis; studies will be divided (where possible) into primary vs. secondary RP. Studies which combine both primary and secondary will only separate into two sub-groups if data is available to do so. Evidence suggests that primary and secondary RP may have different underlying pathogenesis, with studies reporting clear differences in onset, course and prognosis between the groups. [18] Second, we will analyse outcomes based on the presence or absence of identified psychological model of behaviour change, comparing theoretically informed interventions with pragmatic interventions. Interventions which are empirically supported ad evidence-based are more likely to be efficacious than their counterparts, therefore sub-division will allow us to identify whether there is a discrepancy and will allow a more meaningful interpretation of findings. Analysis of sub-groups will include aforementioned test of homogeneity to assess potential group effects, findings will be interpreted accordingly and further analysis performed where appropriate.

ETHICS AND DISSEMINATION

The systematic review does not require ethical approval because it will summarise published studies with non-identifiable data. This protocol complies with the 'Preferred Reporting Items for Systematic Review and Meta-Analysis Protocols (PRISMA-P)' guidelines.[9] Findings will be disseminated in peer review articles and reported according to the PRISMA statement.[19]

For peer review only - http://bmjopen.bmj.com/site/about/guidelines.xhtml

Acknowledgments

Elizabeth Sheils for her assistance with study and protocol preparation and administration.

Contribution of authors

All authors, JD, JP and CE were involved in developing the focus of the systematic review, arriving at the final conception of the proposed systematic review through an iterative process. JD produced the first draft of the protocol and supplementary information, coordinated and assimilated comments from JP and CE on the protocol, approved the final version for publication and is the guarantor of the manuscript. JP also critically revised successive drafts of the manuscript, provided expert advice and intellectual input regarding the subject area (Raynaud's Phenomenon) and approved the final version for publication. CE critically revised successive drafts of the manuscript, provided expert advice on systematic review methodology and approved the final version for publication.

Funding statement

This research received no specific grant or financial support from any funding agency in the public, commercial or not-for-profit sectors.

Competing Interests

None to declare.

For peer review only

REFERENCES

1. Johnson JM, Kellogg DL, Jr. Local thermal control of the human cutaneous circulation. *JAppl Physiol*2010;109(4):1229-38

2. Suter LG, Murabito JM, Felson DT, Fraenkel L. The incidence and natural history of Raynaud's phenomenon in the community. *ArthritisRheum*2005;52(4):1259-63

3. Mayes MD, Lacey JV, Jr., Beebe-Dimmer J, Gillespie BW, Cooper B, Laing TJ, et al. Prevalence, incidence, survival, and disease characteristics of systemic sclerosis in a large US population. *ArthritisRheum*2003;48(8):2246-55

4. Freedman RR, Ianni P. Role of cold and emotional stress in Raynaud's disease and scleroderma. *Br MedJ(ClinResEd)*1983;287(6404):1499-502.

5. National Institute for Clinical and Health Excellence (NICE) (2014). Raynaud's phenomenon: Clinical Knowledge Summary. https://cks.nice.org.uk/raynauds-phenomenon#!topicsummary (accessed December 11th 2016).

6. Vermeire E, Hearnshaw H, Van Royen P, et al. Patient adherence to treatment: three decades of research. A comprehensive review. *JClinPharmTher*2001;26(5):331-42

7. Ennis H, Anderson ME, Wilkinson J, Herrick AL. Calcium channel blockers for primary Raynaud's phenomenon. *Cochrane Database SystRev*2014(1)

8. Pope JE. Raynaud's phenomenon (primary). *BMJClinEvid* 2011;2011 ,[published Online First: 2011/03/16].

9. Shamseer Larissa, Moher David, Clarke Mike, Ghersi Davina, Liberati Alessandro, Petticrew Mark et al. Preferred reporting items for systematic review and meta-analysis protocols (PRISMA-P) 2015: elaboration and explanation. *BMJ*2015; 349: g7647

10. Gladue H, Maranian P, Paulus HE, Khanna D. Evaluation of test characteristics for outcome measures used in Raynaud's phenomenon clinical trials. *ArthritisCareRes.*2013Apr1;65(4):630-6.

11. Merkel PA, Herlyn K, Martin RW, Anderson JJ, Mayes MD, Bell P, *et al.* Measuring disease activity and functional status in patients with scleroderma and Raynaud's phenomenon. *ArthritisRheum*2002;46 2410–20.

12. Chambless DL, Hollon SD. Defining empirically supported therapies. *JConsultClinPsychol*1998;66(1):7.

For peer review only - http://bmjopen.bmj.com/site/about/guidelines.xhtml

For peer review only

13. Higgins JPT, Green S. Cochrane handbook for systematic reviews of interventions: John Wiley & Sons 2011.

14. Guyatt GH, Oxman AD, Vist GE, et al., GRADE Working Group. GRADE: an emerging consensus on rating quality of evidence and strength of recommendations. *BMJ*2008;336:924–6.

15. Cochrane Pain, Palliative & Supportive Care Review Group (2011) Authoring or assessing a Cochrane Protocol, Review, or Review Update. http://papas.cochrane.org/papas-documents (accessed 22 January 2013).

16. Johnston BC, Patrick' DL, Devji' T, Bingham C, Busse JW, Christensen R et al. *Patient-Reported Outcomes: Cochrane Handbook*, vol. 4, in press.

17. Jacobson NS, Truax P. Clinical significance: a statistical approach to defining meaningful change in psychotherapy research. *JConsultClinPsychol*1991;59(1):12.

18. Herrick AL. Pathogenesis of Raynaud's phenomenon. *Rheumatology*2005;44(5):587-96.

19. Moher D, Liberati A, Tetzlaff J, et al. Preferred reporting items for systematic reviews and meta-analyses: the PRISMA statement. *IntJSurg* 2010;8:336–41.

For peer review only

Supplementary Table 1: PRISMA-P Checklist with manuscript page number reference

Section and topic	Page	Item No	Checklist item
**ADMINISTRATIVE INFORMATION**			
Title:			
Identification	1,2,3	1a	Identify the report as a protocol of a systematic review
Update	NA	1b	If the protocol is for an update of a previous systematic review, identify as such
Registration	2,4	2	If registered, provide the name of the registry (such as PROSPERO) and registration number
Authors:			
Contact	1	3a	Provide name, institutional affiliation, e-mail address of all protocol authors; provide physical mailing address of corresponding author
Contributions	9	3b	Describe contributions of protocol authors and identify the guarantor of the review
Amendments	NA	4	If the protocol represents an amendment of a previously completed or published protocol, identify as such and list changes; otherwise, state plan for documenting important protocol amendments
Support:			
Sources	9	5a	Indicate sources of financial or other support for the review
Sponsor	9	5b	Provide name for the review funder and/or sponsor
Role of sponsor or funder	9	5c	Describe roles of funder(s), sponsor(s), and/or institution(s), if any, in developing the protocol
**INTRODUCTION**			
Rationale	3	6	Describe the rationale for the review in the context of what is already known
Objectives	3	7	Provide an explicit statement of the question(s) the review will address with reference to participants, interventions, comparators, and outcomes (PICO)
**METHODS**			
Eligibility criteria	4	8	Specify the study characteristics (such as PICO, study design, setting, time frame) and report characteristics (such as years considered, language, publication status) to be used as criteria for eligibility for the review
Information sources	5 Online sup 2	9	Describe all intended information sources (such as electronic databases, contact with study authors, trial registers or other grey literature sources) with planned dates of coverage
Search strategy	5 Online sup 2	10	Present draft of search strategy to be used for at least one electronic database, including planned limits, such that it could be repeated
Study records:			
Data management	5	11a	Describe the mechanism(s) that will be used to manage records and data throughout the review
Selection process	5	11b	State the process that will be used for selecting studies (such as two independent reviewers) through each phase of the review (that is, screening, eligibility and inclusion in meta-analysis)
Data collection process	5	11c	Describe planned method of extracting data from reports (such as piloting forms, done independently, in duplicate), any processes for obtaining and confirming data from investigators

For peer review only

Data items	4, 5	12	List and define all variables for which data will be sought (such as PICO items, funding sources), any pre-planned data assumptions and simplifications
Outcomes and prioritization	4, 5	13	List and define all outcomes for which data will be sought, including prioritization of main and additional outcomes, with rationale
Risk of bias in individual studies	6	14	Describe anticipated methods for assessing risk of bias of individual studies, including whether this will be done at the outcome or study level, or both; state how this information will be used in data synthesis
Data synthesis	7	15a	Describe criteria under which study data will be quantitatively synthesised
	7	15b	If data are appropriate for quantitative synthesis, describe planned summary measures, methods of handling data and methods of combining data from studies, including any planned exploration of consistency (such as I^2, Kendall's τ)
	7, 8	15c	Describe any proposed additional analyses (such as sensitivity or subgroup analyses, meta-regression)
	7	15d	If quantitative synthesis is not appropriate, describe the type of summary planned
Meta-bias(es)	NA	16	Specify any planned assessment of meta-bias(es) (such as publication bias across studies, selective reporting within studies)
Confidence in cumulative evidence	6	17	Describe how the strength of the body of evidence will be assessed (such as GRADE)

For peer review only

Supplementary Table 2 & 3: Search Strategy

Search engines used: Medline, Embase, PsychINFO, Cochrane (see table 1 and 2 for search terms and strings for respective search engines).

Table 2: Medline (PubMed), PsychINFO, Cochrane Search terms

Number	Strategy
#1	"raynaud's disease" OR Raynaud* OR "Raynaud's phenomenon"
#2	cognitive therap* OR behavior therap* OR Biofeedback OR Psychotherap* OR "Clinical psychology" OR self-management OR non-pharmacological OR "Cognitive Behavioral Therapy" OR "Cold avoidance" OR "behavior* medicine" OR "psychological intervention*" OR "psychoeducation" OR "education"
#3	#1 AND #2

Note. Searching for above terms within 'title and article' and only clinical trials; * = truncation to find plurals, alternative spellings and related concepts.

Table 3: Embase Search Strategy

Number	Strategy
#1	'raynaud phenomenon'
#2	'cognitive therapy' OR 'behavior therapy' OR 'feedback system' OR psychotherapy OR 'clinical psychology' OR 'self care' OR non-pharmacological OR 'cognitive therapy' OR 'cold avoidance' OR 'behavioral medicine' OR 'psychological intervention' OR 'psychoeducation' OR 'education'
#3	#1 AND #2

Note. Searching for above terms within 'title and article' and only clinical trials; * = truncation to find plurals, alternative spellings and related concepts.

For peer review only

Supplementary Table 4: Data extraction

Systematic review of behaviour change interventions for Raynaud's Phenomenon: Data Extraction Form

**Citation** (include live link)	
**Retrieval information (date/location)**	

Eligibility criteria

**Adults with RP (primary or secondary)**	
**Randomised controlled trial**	
**At least one active behaviour change intervention and control**	

**JD**
**JP**
**CE**

Study Details

**Patient population**	**Primary RP (n)**	**Systemic sclerosis (n)**	**Other secondary RP (describe in box below)**
**Diagnostic criteria used**			
**Year of publication**			
**Study design**			
**Format of Intervention**			
**Study setting / country**			
**Sample characteristics (incl. size & subgroup)**			
**Gender**			
**Age**			
**Ethnicity**			
**Active treatment Interventions**			
**Pre-defined behaviour change model/theory**	Yes / No	Please state:	
**Control intervention**			
**Duration of study**			
**Primary end-points**			
**Secondary end-points**			
**No. treatment sessions**			
**Level of therapist training**			
**Integrity of the intervention checked?**			
**Adverse events**			
**Outcome/results:**			
**Frequency of attacks**	**Duration of RP attacks**	**Severity of RP attacks**	

**Comments**

Supplementary Table 5: Data extraction – risk of bias

For peer review only

Risk of bias assessment

Study Validity Domains	Assessment	Comments
1. Sequence Generation: Was the allocation sequence adequately controlled?	Yes ☐<br>No ☐<br>Unclear ☐	
2. Allocation concealment: Was the sequence generation adequately concealed before group assignments?	Yes ☐<br>No ☐<br>Unclear ☐	
3. Blinding of participants and personnel: Was knowledge of the allocated interventions adequately hidden from the participants and personnel after the participants were assigned to respective groups?	Yes ☐<br>No ☐<br>Unclear ☐	
4. Blinding of outcome assessors: Was knowledge of the allocated interventions adequately hidden from the outcome assessors after participants were assigned to respective groups	Yes ☐<br>No ☐<br>Unclear ☐	
5. Incomplete outcome data: Were incomplete outcome data adequately addressed?	Yes ☐<br>No ☐<br>Unclear ☐	
6. Selective outcome reporting: Are reports of the study free of suggestion of selective outcome reporting?	Yes ☐<br>No ☐<br>Unclear ☐	
7. Other sources of bias: Was the study apparently free of other problems that could put it at a risk of bias?	Yes ☐<br>No ☐<br>Unclear ☐	

For peer review only

Research Checklist:

PRISMA-P Checklist with manuscript page number reference

Section and topic	Page	Item No	Checklist item
**ADMINISTRATIVE INFORMATION**			
Title:			
Identification	1,2,3	1a	Identify the report as a protocol of a systematic review
Update	NA	1b	If the protocol is for an update of a previous systematic review, identify as such
Registration	2,4	2	If registered, provide the name of the registry (such as PROSPERO) and registration number
Authors:			
Contact	1	3a	Provide name, institutional affiliation, e-mail address of all protocol authors; provide physical mailing address of corresponding author
Contributions	9	3b	Describe contributions of protocol authors and identify the guarantor of the review
Amendments	NA	4	If the protocol represents an amendment of a previously completed or published protocol, identify as such and list changes; otherwise, state plan for documenting important protocol amendments
Support:			
Sources	9	5a	Indicate sources of financial or other support for the review
Sponsor	9	5b	Provide name for the review funder and/or sponsor
Role of sponsor or funder	9	5c	Describe roles of funder(s), sponsor(s), and/or institution(s), if any, in developing the protocol
**INTRODUCTION**			
Rationale	3	6	Describe the rationale for the review in the context of what is already known
Objectives	3	7	Provide an explicit statement of the question(s) the review will address with reference to participants, interventions, comparators, and outcomes (PICO)
**METHODS**			
Eligibility criteria	4	8	Specify the study characteristics (such as PICO, study design, setting, time frame) and report characteristics (such as years considered, language, publication status) to be used as criteria for eligibility for the review
Information sources	5 Online sup 2	9	Describe all intended information sources (such as electronic databases, contact with study authors, trial registers or other grey literature sources) with planned dates of coverage
Search strategy	5 Online sup 2	10	Present draft of search strategy to be used for at least one electronic database, including planned limits, such that it could be repeated
Study records:			
Data management	5	11a	Describe the mechanism(s) that will be used to manage records and data throughout the review
Selection process	5	11b	State the process that will be used for selecting studies (such as two independent reviewers) through each phase of the review (that is, screening, eligibility and inclusion in meta-analysis)
Data collection	5	11c	Describe planned method of extracting data from reports (such as

For peer review only

process			piloting forms, done independently, in duplicate), any processes for obtaining and confirming data from investigators
Data items	4, 5	12	List and define all variables for which data will be sought (such as PICO items, funding sources), any pre-planned data assumptions and simplifications
Outcomes and prioritization	4, 5	13	List and define all outcomes for which data will be sought, including prioritization of main and additional outcomes, with rationale
Risk of bias in individual studies	6	14	Describe anticipated methods for assessing risk of bias of individual studies, including whether this will be done at the outcome or study level, or both; state how this information will be used in data synthesis
Data synthesis	7	15a	Describe criteria under which study data will be quantitatively synthesised
	7	15b	If data are appropriate for quantitative synthesis, describe planned summary measures, methods of handling data and methods of combining data from studies, including any planned exploration of consistency (such as I^2, Kendall's τ)
	7, 8	15c	Describe any proposed additional analyses (such as sensitivity or subgroup analyses, meta-regression)
	7	15d	If quantitative synthesis is not appropriate, describe the type of summary planned
Meta-bias(es)	NA	16	Specify any planned assessment of meta-bias(es) (such as publication bias across studies, selective reporting within studies)
Confidence in cumulative evidence	6	17	Describe how the strength of the body of evidence will be assessed (such as GRADE)

For peer review only

Behaviour change interventions for the management of Raynaud's phenomenon: a systematic review protocol

Journal:	*BMJ Open*
Manuscript ID	bmjopen-2017-017039.R1
Article Type:	Protocol
Date Submitted by the Author:	29-May-2017
Complete List of Authors:	Daniels, Jo; University of Bath, Dept. Psychology<br>Pauling, John; University of Bath , Dept. Pharmacy and Pharmacology ; Royal National Hospital For Rheumatic Diseases NHS Foundation Trust, Rheumatology Department<br>Eccleston, Christopher; University of Bath, Centre for Pain Research
**Primary Subject Heading**:	Rheumatology
Secondary Subject Heading:	Rheumatology
Keywords:	RAYNAUD'S PHENOMENON, behaviour change, Systematic review

SCHOLARONE™ Manuscripts

Behaviour change interventions for the management of Raynaud's phenomenon: a systematic review protocol

Daniels, J. [1] Pauling, J.D.[2,3] & Eccleston, C.[4]

Word count (excluding references and abstract): 2191

[1] Department of Psychology, The University of Bath, Claverton Down, Bath, BA2 7AY, United Kingdom.

[2] Department of Rheumatology, Royal National Hospital for Rheumatic Diseases, Upper Borough Walls, Bath BA1 1RL

[3]Department of Pharmacy and Pharmacology, The University of Bath, Claverton Down, Bath, BA2 7AY, United Kingdom.

[4] Centre for Pain Research, Department for Health, The University of Bath, Bath, BA2 7AY, United Kingdom.

*Requests for reprints should be addressed to Dr Jo Daniels, University of Bath, Department of Psychology, Claverton Down, Bath BA2 7AY, UK, t: 01225385506 e: j.daniels@bath.ac.uk

For peer review only - http://bmjopen.bmj.com/site/about/guidelines.xhtml

ABSTRACT

Introduction: Raynaud's Phenomenon (RP) describes excessive peripheral vasospasm to cold exposure and/or emotional stress. RP episodes are associated with digital colour changes, pain and reduced quality of life. Pharmacological interventions are of low to moderate efficacy and often result in adverse effects such as facial flushing and headaches. Recommended lifestyle and behavioural interventions have not been evaluated. The objectives of the proposed systematic review are to assess the comparative safety and efficacy of behaviour change interventions for RP and identify what we can learn to inform future interventions.

Methods and analysis: Studies eligible for inclusion include randomised controlled trials (RCTs) testing a behaviour change interventions with control comparator. A comprehensive search strategy will include peer-review and grey literature up until 30th April 2017. Search databases will include Medline, Embase, PsychINFO and Cochrane. Initial sifting, eligibility, data extraction, risk of bias and quality assessment will be subject to review by two independent reviewers with a third reviewer resolving discrepancies. Risk of bias assessment will be performed using Cochrane risk of bias assessment tool with quality of evidence assessed using GRADE. A meta-analysis will be performed if there are sufficient data. Two subgroup analyses are planned: primary vs. secondary RP outcomes; comparison of theoretically-informed interventions with pragmatic interventions.

Ethics and dissemination: This review does not require ethical approval as it will summarise published studies with non-identifiable data. This protocol complies with 'Preferred Reporting Items for Systematic Review and Meta-Analysis Protocols (PRISMA-P) guidelines. Findings will be disseminated in peer-reviewed articles and reported according to PRISMA. This review will make a significant contribution to the management of RP where no review of behaviour-change interventions currently exist. The synopsis and protocol for the proposed systematic review is registered in the International Prospective Register of Systematic Reviews (registration number CRD42017049643).

For peer review only

STRENGTHS AND LIMITATIONS

- This study will offer the first review of behaviour-change interventions for the treatment of Raynaud 's phenomenon, making a significant contribution to our current understanding of treatment options and interventions for this common medical complaint.
- Risk of bias assessment will be performed using Cochrane risk of bias assessment tool with quality of evidence assessed using GRADE. We will also assess for quality of each psychotherapeutic intervention, further comparing theoretically-underpinned and pragmatic interventions.
- We will restrict our review to randomised controlled trials only, which may exclude studies of potential interest. However, we aim to evaluate efficacy of high quality interventions and use the outcomes of this review to inform future high quality interventions for Raynaud's Phenomenon, therefore we believe this exclusion is appropriate. We intend to discuss noteworthy non-randomised trials or observational studies, however this will not be part of our specified search.

For peer review only

Key words: Raynaud's Phenomenon, behaviour change, systematic review

For peer review only - http://bmjopen.bmj.com/site/about/guidelines.xhtml

INTRODUCTION

Peripheral vasoconstriction of thermoregulatory pre-capillary arterioles and arteriovenous anastomoses is a normal physiological response to cold exposure designed to preserve normal core temperature.[1] Raynaud's Phenomenon (RP) describes excessive peripheral vasospasm to cold exposure and/or emotional stress. Attacks of RP are associated with characteristic digital colour changes (reflecting blood oxygenation and tissue perfusion). Tissue ischaemia (and subsequent reperfusion) during attacks of RP results in pain and paraesthesia causing distress, loss of hand function and reduced quality of life. RP is common, affecting approximately 10% of people.[2] The majority of RP sufferers have a functional vasospastic disorder that, whilst intrusive, is otherwise benign in nature (termed primary RP). Digital perfusion is generally normal in between attacks. In other circumstances, symptoms of RP occur as a result of disturbed digital tissue perfusion related to separate underlying pathology (termed secondary RP). Important causes of secondary RP are the autoimmune rheumatic diseases such as systemic sclerosis (SSc). In SSc, endothelial damage leads to an obliterative microangiopathy, characterised by structural changes to the vessel wall and near permanent tissue ischaemia. SSc, despite being rare with an estimated prevalence of 250/million,[3] is often used as the focus of RP research. Cold exposure is the most frequent precipitating factor, although emotional stress may account for up to 1/3 of RP attacks.[4]

Behavioural approaches to RP management include lifestyle and habit reversal interventions such as avoiding cold exposure, conserving heat loss, smoking cessation, increasing exercise, and reducing stress levels.[5] It is recognised that adherence with interventions of this nature can be poor with estimates of 30-50% of patients demonstrating poor compliance, regardless of condition, expected outcome, or setting.[6] Pharmacological interventions to induce vasodilation and prevent excessive vasoconstriction of the digital microvasculature also have a role in RP management but the efficacy of such treatments is modest in terms of frequency and severity of RP attacks.[7] Furthermore, pharmacological intervention to promote vasodilation often results in adverse effects such as facial flushing, headaches, fluid retention, dizziness, and palpitations.[8] Despite the perceived importance of non-pharmacological interventions, comparative efficacy, adoption and compliance with lifestyle interventions has not been fully evaluated.[5] A number of behavioural interventions have been tested for RP but the comparative efficacy of a range of interventions within different disease populations (primary and secondary RP) has not previously been the focus of a systematic review. The specific objectives of the proposed systematic review are to assess the comparative safety and efficacy of a range of behavioural interventions for the management and treatment of symptoms

For peer review only - http://bmjopen.bmj.com/site/about/guidelines.xhtml

associated with primary and secondary RP. The secondary objective is to identify what we can learn from the studies reviewed to inform future behaviour change interventions for RP.

METHODS AND ANALYSIS

Reporting of protocol and review registration.

This protocol follows PRIMSA-P guidelines[9] for the reporting of a systematic review protocol (see Online Supplementary table 1). The synopsis and protocol for this systematic review is registered in the International Prospective Register of Systematic Reviews (http://www.crd.york.ac.uk/PROSPERO)[10]. Reference was also made to the generic Cochrane protocol for pharmacological interventions for the treatment of RP.[11]

Study Selection

Randomised Controlled Trials (RCTs) testing one or more active treatment interventions with a control comparator arm will be included. Both individual and cluster randomisation will be included providing cluster sites meet other inclusion criteria. Non-randomised controlled trials and other intervention study designs will not be included. Blinding in non-pharmacological interventions is not always possible or relevant. Non-blinded studies will be included.

Participants

Adults (18 years or older) with a diagnosis of Raynaud's Phenomenon (primary or secondary). Mixed samples studies will be included. The search will be limited to studies with humans

Interventions

We define behaviour change interventions as interventions which target symptomatic relief of RP through directed or advised change in patient determined behaviour. We are particularly interested in studies consistent with NICE guidelines for RP,[5] however we purposefully include all interventions which are designed to change behaviour to improve symptoms. Behaviour change interventions are described variably and may use the following adjectives, all of which will be used in the search strategy: behavio(u)ral therapy, cognitive therapy, education, psychoeducation, biofeedback, clinical psychology, psychological, psychotherapy, self-management, cognitive behavio(u)r therapy, and behavioural medicine.

Outcome measures

There is no consensus over the domains of measurement or measurement technology.[12]

For peer review only - http://bmjopen.bmj.com/site/about/guidelines.xhtml

For peer review only

Assessment of RP is largely reliant on patient-reported outcomes, typically captured using instruments that monitor RP symptoms over 1-2 weeks (such as the Raynaud's Condition Score [RCS] diary)[13]. The primary outcomes measures chosen for our analyses mirror those adopted in a recent generic systematic review protocol for RP[11] and include; the severity/impact of RP episodes assessed using visual analogue scales (VAS), Likert scales, or the RCS (either at a single time point using a standardised recall period) or as an average daily score (obtained from the RCS diary or equivalent from RP symptom diary); frequency of RP attacks (adopted from the RCS diary or equivalent symptom diary approach) reported as average daily or weekly frequency of RP attacks; duration of RP attacks (adopted from the RCS diary or equivalent symptom diary approach) reported as the average daily duration of RP attacks over 1-2 weeks; pain assessed using a VAS or Likert scale (reporting intensity of pain during RP attacks) and patient assessment of disability due to RP/interference on daily activities e.g. the Scleroderma Health Assessment Questionnaire (HAQ) Raynaud's phenomenon VAS or equivalent. Adverse events (hospitalization/death) and withdrawals from study will also be included within our primary outcomes.

Secondary outcomes will include physician global assessment of severity/impact of RP; patient global assessment of function/disability secondary to RP (e.g. the HAQ score); change in digital ulceration (positive/negative); treatment preference and general improvement (self-reported overall improvement). Outcome data on anxiety and depression will also be collated where available. Most RP clinical trials involve assessments over 1 or more weeks. Sensitivity analyses shall be undertaken if the analyses include trials with marked differences in duration of treatment/assessment.

Search Strategy

A comprehensive search strategy has been created, which includes searching of grey-literature. Four sources of peer-review databases will be used: Medline, Embase, PsychINFO and Cochrane. The search will have no language or publication date restrictions, seeking to translate where necessary. Studies must be fully published at the time of search; early view online publications will be included. A draft search strategy is included in the online supplementary table 2 & 3. Endnote and Covidence software (covidence.org) will be used for data management.

Selection of Studies

All studies generated by the search will be screened by two review authors for eligibility. Full texts will be retrieved for those which meet eligibility, which will then be rated independently by two

For peer review only - http://bmjopen.bmj.com/site/about/guidelines.xhtml

review authors using a pre-specified data extraction form (online supplementary tables 4 & 5). Bibliographies of included studies will also be searched for inclusion of relevant studies, which will then be subject to independent review of eligibility. Discrepancies will be reviewed, with resolution through discussion and consultation of a third author reviewer. Level of agreement will be calculated.

Data collection process

All eligible studies will be subject to independent data extraction by two review authors. A data extraction form has been developed and piloted to extract the following: full citation including author and year of publication; retrieval information(date/database); patient population; diagnostic criteria used for RP diagnosis; study design; intervention(s); study setting/county (including language); format of intervention (e.g. group/individual/internet); sample size and description; age/gender/ethnicity/ intervention(s); duration of study; number of treatment sessions; primary endpoints; secondary outpoints; frequency, duration and severity of RP attacks; outcome/results; comments, adverse events.

We will also collect information pertaining to the quality of psychotherapeutic interventions: (a) reference to a theoretical model (b) level of therapist training (c) whether the integrity of the intervention was checked. These criteria are drawn from an authoritative review of empirically supported psychotherapies .[14] We will attempt to retrieve missing data with study authors.

The data extraction form will feature a brief eligibility check box with core inclusion criteria: adults with RP; behaviour change intervention with at least one comparator; RCT; RCS or equivalent. All criteria need to be present in order to demonstrate eligibility.

Risk of Bias

Two review authors will independently assess risk of bias on an outcome and study level using the Cochrane risk of bias assessment tool. Unresolved discrepancies will be reviewed by the third review author. We will assess risk of bias according to the following dimensions: random sequence generation (adequate description and method of participant allocation in accordance with standard randomisation); allocation concealment (adequate concealment of group assignment to prevent selection bias); blinding of participants and personnel (adequacy of measures taken to prevent performance bias and conceal group assignment); blinding outcome assessors (adequacy of measures taken to prevent detection bias and conceal group assignment to outcome assessors); incomplete data (adequacy of the management of missing data and potential implications for bias); selective outcome reporting (reporting bias relating to the consistency between pre-specified and

For peer review only - http://bmjopen.bmj.com/site/about/guidelines.xhtml

reported outcomes); other sources of bias (other concerns not covered elsewhere but may lead to a risk of bias). All eligible studies will be rated as high low, or unclear (risk of bias), on each of these dimensions, culminating in an overall risk of bias (high/low/unclear) in accordance with the Cochrane handbook for systematic reviews of interventions.[15] Funnel plots will be used to assess bias and will be stratified based on bias assessment, if considered useful and appropriate (>10 studies).

Quality of evidence

We will assess the quality of the evidence, and so our confidence in any estimates of effect using GRADE: (in)consistency of effect, imprecision, indirectness and publication bias. [16] If possible we will construct a GRADE summary of findings table, using the seven primary outcomes. When possible we will express dichotomous outcomes (e.g. no less than mild pain defined as <30 on a 100 point scale,[17] or 50% reduction in the number of episodes, as NNT, or minimal importance clinical differences.[18]

ANALYSIS

We will perform a meta-analysis if there are sufficient data, and it is clinically sensible.

Effect sizes will be calculated at post-test between therapies for each comparison with control (using hedges g) by subtracting average score of active arm from average of control arm and dividing by pooled standard deviation. We will also calculate mean/median differences from baseline to post treatment for each invention and mean differences of change in measures associated with primary outcomes (where appropriate).

The review outcomes are likely to be reported in both dichotomous and continuous data. For dichotomous data we will report the results as summary of risk ratios and confidence interval (CI) levels of 95%, pooling those data with identical outcomes and interventions. For continuous data, standardised mean difference and 95% CI will be used due to likelihood of discrepant scales used in the study. The Cochrane handbook will be used for translation of outcomes of scales.

Heterogeneity will be assessed on the basis of study design, participants, interventions and outcomes and will be assessed using forest plots (using 10% level). Higgins I^2 statistic, X^2, and visual inspection of forest plots will be used to assess homogeneity with thresholds of 50-75% for moderate heterogeneity and 75% for significant heterogeneity. We anticipate the use of random-

For peer review only - http://bmjopen.bmj.com/site/about/guidelines.xhtml

effects meta-analysis due to the likely heterogeneity of participant and outcomes data however fixed models will be used where appropriate.

In the absence of a meta-analysis, a narrative (descriptive) analysis of primary and secondary outcomes will be provide and will include: 1. Aggregated data on mean daily frequency/severity/duration of episodes, patient global assessment of disability and changes on RCS scores. 2. Analysis of reported design or intervention flaws in behaviour change interventions 3. Analysis of reported considerations as regards of future behaviour change interventions.

Due to the lack of reliable endpoints in RP treatment trials, we will specify minimally important differences (MIDs) in measures to allow clinically meaningful interpretation of change on standardised measures: 15-point difference on RCS, 20% difference on VAS and likert scales (based previous estimates in RP [12]) use of reliable change index (RCI)[19] for other standardised measures.

Planned sub-group analysis.

Two subgroup analyses are planned based on specific clinical hypotheses. First, we will analyse outcomes by diagnosis; studies will be divided (where possible) into primary vs. secondary RP. Studies which combine both primary and secondary will only separate into two sub-groups if data is available to do so. Evidence suggests that primary and secondary RP may have different underlying pathogenesis, with studies reporting clear differences in onset, course and prognosis between the groups. [20] Second, we will analyse outcomes based on the presence or absence of identified psychological model of behaviour change, comparing theoretically informed interventions with pragmatic interventions. Interventions which are empirically supported ad evidence-based are more likely to be efficacious than their counterparts, therefore sub-division will allow us to identify whether there is a discrepancy and will allow a more meaningful interpretation of findings. Analysis of sub-groups will include aforementioned test of homogeneity to assess potential group effects, findings will be interpreted accordingly and further analysis performed where appropriate.

ETHICS AND DISSEMINATION

The systematic review does not require ethical approval because it will summarise published studies with non-identifiable data. This protocol complies with the 'Preferred Reporting Items for Systematic Review and Meta-Analysis Protocols (PRISMA-P)' guidelines.[9] Findings will be disseminated in peer review articles and reported according to the PRISMA statement.[21]

For peer review only - http://bmjopen.bmj.com/site/about/guidelines.xhtml

For peer review only

For peer review only - http://bmjopen.bmj.com/site/about/guidelines.xhtml

Acknowledgments

Elizabeth Sheils for her assistance with study and protocol preparation and administration.

Contribution of authors

All authors, JD, JP and CE were involved in developing the focus of the systematic review, arriving at the final conception of the proposed systematic review through an iterative process. JD produced the first draft of the protocol and supplementary information, coordinated and assimilated comments from JP and CE on the protocol, approved the final version for publication and is the guarantor of the manuscript. JP also critically revised successive drafts of the manuscript, provided expert advice and intellectual input regarding the subject area (Raynaud's Phenomenon) and approved the final version for publication. CE critically revised successive drafts of the manuscript, provided expert advice on systematic review methodology and approved the final version for publication.

Funding statement

This research received no specific grant or financial support from any funding agency in the public, commercial or not-for-profit sectors.

Competing Interests

None to declare.

For peer review only

REFERENCES

1. Johnson JM, Kellogg DL, Jr. Local thermal control of the human cutaneous circulation. *JAppl Physiol*2010;109(4):1229-38

2. Suter LG, Murabito JM, Felson DT, Fraenkel L. The incidence and natural history of Raynaud's phenomenon in the community. *ArthritisRheum*2005;52(4):1259-63

3. Mayes MD, Lacey JV, Jr., Beebe-Dimmer J, Gillespie BW, Cooper B, Laing TJ, et al. Prevalence, incidence, survival, and disease characteristics of systemic sclerosis in a large US population. *ArthritisRheum*2003;48(8):2246-55

4. Freedman RR, Ianni P. Role of cold and emotional stress in Raynaud's disease and scleroderma. *Br MedJ(ClinResEd)*1983;287(6404):1499-502.

5. National Institute for Clinical and Health Excellence (NICE) (2014). Raynaud's phenomenon: Clinical Knowledge Summary. https://cks.nice.org.uk/raynauds-phenomenon#!topicsummary (accessed December 11th 2016).

6. Vermeire E, Hearnshaw H, Van Royen P, et al. Patient adherence to treatment: three decades of research. A comprehensive review. *JClinPharmTher*2001;26(5):331-42

7. Ennis H, Anderson ME, Wilkinson J, Herrick AL. Calcium channel blockers for primary Raynaud's phenomenon. *Cochrane Database SystRev*2014(1)

8. Pope JE. Raynaud's phenomenon (primary). *BMJClinEvid* 2013;2013: 1119

9. Shamseer L, Moher D, Clarke M, Ghersi D, Liberati A, Petticrew M et al. Preferred reporting items for systematic review and meta-analysis protocols (PRISMA-P) 2015: elaboration and explanation. *BMJ*2015; 349: g7647

10. Daniels J, Pauling J Eccleston C. Behaviour change interventions for the management of Raynaud's phenomenon: a systematic review protocol. PROSPERO: International prospective register of systematic reviews. 2017. Available from: https://www.crd.york.ac.uk/PROSPERO/display_record.asp?ID=CRD42017049643

11. Pope J, Rirash F, Tingey PC, Harding SE, Maxwell LJ, Tanjong Ghogomu E, Tugwell P, Wells GA. Drug interventions versus placebo for the treatment of Raynaud's phenomenon: generic protocol. *Cochrane Database of Systematic Reviews* 2015, Issue 8. Art. No.: CD011813.

For peer review only - http://bmjopen.bmj.com/site/about/guidelines.xhtml

12. Gladue H, Maranian P, Paulus HE, Khanna D. Evaluation of test characteristics for outcome measures used in Raynaud's phenomenon clinical trials. *ArthritisCareRes.*2013Apr1;65(4):630-6.

13. Merkel PA, Herlyn K, Martin RW, Anderson JJ, Mayes MD, Bell P, *et al.* Measuring disease activity and functional status in patients with scleroderma and Raynaud's phenomenon. *ArthritisRheum*2002;46 2410–20.

14. Chambless DL, Hollon SD. Defining empirically supported therapies. *JConsultClinPsychol*1998;66(1):7.

15. Higgins JPT, Green S. Cochrane handbook for systematic reviews of interventions: John Wiley & Sons 2011.

16. Guyatt GH, Oxman AD, Vist GE, et al., GRADE Working Group. GRADE: an emerging consensus on rating quality of evidence and strength of recommendations. *BMJ*2008;336:924–6.

17. Cochrane Pain, Palliative & Supportive Care Review Group (2011) Authoring or assessing a Cochrane Protocol, Review, or Review Update. http://papas.cochrane.org/papas-documents (accessed 22 January 2013).

18. Johnston BC, Patrick DL, Devji T, Bingham C, Busse JW, Christensen R et al. *Patient-Reported Outcomes: Cochrane Handbook*, vol. 4, in press.

19. Jacobson NS, Truax P. Clinical significance: a statistical approach to defining meaningful change in psychotherapy research. *JConsultClinPsychol*1991;59(1):12.

20. Herrick AL. Pathogenesis of Raynaud's phenomenon. *Rheumatology*2005;44(5):587-96.

21. Moher D, Liberati A, Tetzlaff J, et al. Preferred reporting items for systematic reviews and meta-analyses: the PRISMA statement. *IntJSurg* 2010;8:336–41.

For peer review only

For peer review only - http://bmjopen.bmj.com/site/about/guidelines.xhtml

Supplementary Table 1: PRISMA-P Checklist with manuscript page number reference

Section and topic	Page	Item No	Checklist item
**ADMINISTRATIVE INFORMATION**			
Title:			
Identification	1,2,3	1a	Identify the report as a protocol of a systematic review
Update	NA	1b	If the protocol is for an update of a previous systematic review, identify as such
Registration	2,4	2	If registered, provide the name of the registry (such as PROSPERO) and registration number
Authors:			
Contact	1	3a	Provide name, institutional affiliation, e-mail address of all protocol authors; provide physical mailing address of corresponding author
Contributions	9	3b	Describe contributions of protocol authors and identify the guarantor of the review
Amendments	NA	4	If the protocol represents an amendment of a previously completed or published protocol, identify as such and list changes; otherwise, state plan for documenting important protocol amendments
Support:			
Sources	9	5a	Indicate sources of financial or other support for the review
Sponsor	9	5b	Provide name for the review funder and/or sponsor
Role of sponsor or funder	9	5c	Describe roles of funder(s), sponsor(s), and/or institution(s), if any, in developing the protocol
**INTRODUCTION**			
Rationale	3	6	Describe the rationale for the review in the context of what is already known
Objectives	3	7	Provide an explicit statement of the question(s) the review will address with reference to participants, interventions, comparators, and outcomes (PICO)
**METHODS**			
Eligibility criteria	4	8	Specify the study characteristics (such as PICO, study design, setting, time frame) and report characteristics (such as years considered, language, publication status) to be used as criteria for eligibility for the review
Information sources	5 Online sup 2	9	Describe all intended information sources (such as electronic databases, contact with study authors, trial registers or other grey literature sources) with planned dates of coverage
Search strategy	5 Online sup 2	10	Present draft of search strategy to be used for at least one electronic database, including planned limits, such that it could be repeated
Study records:			
Data management	5	11a	Describe the mechanism(s) that will be used to manage records and data throughout the review
Selection process	5	11b	State the process that will be used for selecting studies (such as two independent reviewers) through each phase of the review (that is, screening, eligibility and inclusion in meta-analysis)
Data collection process	5	11c	Describe planned method of extracting data from reports (such as piloting forms, done independently, in duplicate), any processes for obtaining and confirming data from investigators

For peer review only

Data items	4, 5	12	List and define all variables for which data will be sought (such as PICO items, funding sources), any pre-planned data assumptions and simplifications
Outcomes and prioritization	4, 5	13	List and define all outcomes for which data will be sought, including prioritization of main and additional outcomes, with rationale
Risk of bias in individual studies	6	14	Describe anticipated methods for assessing risk of bias of individual studies, including whether this will be done at the outcome or study level, or both; state how this information will be used in data synthesis
Data synthesis	7	15a	Describe criteria under which study data will be quantitatively synthesised
	7	15b	If data are appropriate for quantitative synthesis, describe planned summary measures, methods of handling data and methods of combining data from studies, including any planned exploration of consistency (such as I^2, Kendall's τ)
	7, 8	15c	Describe any proposed additional analyses (such as sensitivity or subgroup analyses, meta-regression)
	7	15d	If quantitative synthesis is not appropriate, describe the type of summary planned
Meta-bias(es)	NA	16	Specify any planned assessment of meta-bias(es) (such as publication bias across studies, selective reporting within studies)
Confidence in cumulative evidence	6	17	Describe how the strength of the body of evidence will be assessed (such as GRADE)

For peer review only

Supplementary Table 2 & 3: Search Strategy

Search engines used: Medline, Embase, PsychINFO, Cochrane (see table 1 and 2 for search terms and strings for respective search engines).

Table 2: Medline (PubMed), PsychINFO, Cochrane Search terms

Number	Strategy
#1	"raynaud's disease" OR Raynaud* OR "Raynaud's phenomenon"
#2	cognitive therap* OR behavior therap* OR Biofeedback OR Psychotherap* OR "Clinical psychology" OR self-management OR non-pharmacological OR "Cognitive Behavioral Therapy" OR "Cold avoidance" OR "behavior* medicine" OR "psychological intervention*" OR "psychoeducation" OR "education"
#3	#1 AND #2

Note. Searching for above terms within 'title and article' and only clinical trials; * = truncation to find plurals, alternative spellings and related concepts.

Table 3: Embase Search Strategy

Number	Strategy
#1	'raynaud phenomenon'
#2	'cognitive therapy' OR 'behavior therapy' OR 'feedback system' OR psychotherapy OR 'clinical psychology' OR 'self care' OR non-pharmacological OR 'cognitive therapy' OR 'cold avoidance' OR 'behavioral medicine' OR 'psychological intervention' OR 'psychoeducation' OR 'education'
#3	#1 AND #2

Note. Searching for above terms within 'title and article' and only clinical trials; * = truncation to find plurals, alternative spellings and related concepts.

For peer review only

Supplementary Table 4: Data extraction

Systematic review of behaviour change interventions for Raynaud's Phenomenon: Data Extraction Form

Citation (include live link)	
Retrieval information (date/location)	

Eligibility criteria

Adults with RP (primary or secondary)	
Randomised controlled trial	
At least one active behaviour change intervention and control	

JD
JP
CE

Study Details

Patient population	Primary RP (n)	Systemic sclerosis (n)	Other secondary RP (describe in box below)
Diagnostic criteria used			
Year of publication			
Study design			
Format of Intervention			
Study setting / country			
Sample characteristics (incl. size & subgroup)			
Gender			
Age			
Ethnicity			
Active treatment Interventions			
Pre-defined behaviour change model/theory	Yes / No	Please state:	
Control intervention			
Duration of study			
Primary end-points			
Secondary end-points			
No. treatment sessions			
Level of therapist training			
Integrity of the intervention checked?			
Adverse events			
Outcome/results:			
Frequency of attacks	Duration of RP attacks	Severity of RP attacks	

Comments

For peer review only

Supplementary Table 5: Data extraction – risk of bias

Risk of bias assessment

Study Validity Domains	Assessment	Comments
1. Sequence Generation: Was the allocation sequence adequately controlled?	Yes ☐<br>No ☐<br>Unclear ☐	
2. Allocation concealment: Was the sequence generation adequately concealed before group assignments?	Yes ☐<br>No ☐<br>Unclear ☐	
3. Blinding of participants and personnel: Was knowledge of the allocated interventions adequately hidden from the participants and personnel after the participants were assigned to respective groups?	Yes ☐<br>No ☐<br>Unclear ☐	
4. Blinding of outcome assessors: Was knowledge of the allocated interventions adequately hidden from the outcome assessors after participants were assigned to respective groups	Yes ☐<br>No ☐<br>Unclear ☐	
5. Incomplete outcome data: Were incomplete outcome data adequately addressed?	Yes ☐<br>No ☐<br>Unclear ☐	
6. Selective outcome reporting: Are reports of the study free of suggestion of selective outcome reporting?	Yes ☐<br>No ☐<br>Unclear ☐	
7. Other sources of bias: Was the study apparently free of other problems that could put it at a risk of bias?	Yes ☐<br>No ☐<br>Unclear ☐	

For peer review only

Data Extraction Form v4:

JD
JP
CE

Behaviour change interventions for the management of Raynaud's Phenomenon: a systematic review

**Citation** (include live link)	
**Retrieval information (date/location)**	

Eligibility criteria

**Adults with Raynauds (primary or secondary)**	
**Randomised controlled trial**	
**At least one active behaviour change intervention and control**	

Study Details

**Patient population**	**Primary RP (n)**	**Systemic sclerosis (n)**	**Other secondary RP (describe in box below)**
**Diagnostic criteria used**			
**Year of publication**			
**Study design**			
**Format of Intervention**			
**Study setting / country**			
**Sample characteristics (incl. size & subgroup)**			
**Gender**			
**Age**			
**Ethnicity**			
**Active treatment Interventions**			
**Pre-defined behaviour change model/theory**	Yes / No	Please state:	
**Control intervention**			
**Duration of study**			
**Primary end-points**			
**Secondary end-points**			
**No. treatment sessions**			
**Level of therapist training**			
**Integrity of the intervention checked?**			
**Primary Outcomes:**			
**Frequency of attacks**			
**Duration of attacks**			
**Severity of attacks**			
**Pain**			
**Patient assessment of disability**			
**Adverse events**			
**Withdrawal**			
**Secondary Outcomes:**			
**Physician global assessment of severity**			
**Patient global assessment of disability**			

For peer review only

**Change in digital ulceration**	
**Treatment preference**	
**General improvement**	
**Anxiety**	
**Depression**	

**Comments**

Risk of bias assessment

**Study Validity Domains**	**Assessment**	**Comments**
1. Sequence Generation: Was the allocation sequence adequately controlled?	Yes ☐<br>No ☐<br>Unclear ☐	
2. Allocation concealment: Was the sequence generation adequately concealed before group assignments?	Yes ☐<br>No ☐<br>Unclear ☐	
3. Blinding of participants and personnel: Was knowledge of the allocated interventions adequately hidden from the participants and personnel after the participants were assigned to respective groups?	Yes ☐<br>No ☐<br>Unclear ☐	
4. Blinding of outcome assessors: Was knowledge of the allocated interventions adequately hidden from the outcome assessors after participants were assigned to respective groups	Yes ☐<br>No ☐<br>Unclear ☐	
5. Incomplete outcome data: Were incomplete outcome data adequately addressed?	Yes ☐<br>No ☐<br>Unclear ☐	
6. Selective outcome reporting: Are reports of the study free of suggestion of selective outcome reporting?	Yes ☐<br>No ☐<br>Unclear ☐	
7. Other sources of bias: Was the study apparently free of other problems that could put it at a risk of bias?	Yes ☐<br>No ☐<br>Unclear ☐	

For peer review only

Research Checklist:

PRISMA-P Checklist with manuscript page number reference

Section and topic	Page	Item No	Checklist item
**ADMINISTRATIVE INFORMATION**			
Title:			
Identification	1,2,3	1a	Identify the report as a protocol of a systematic review
Update	NA	1b	If the protocol is for an update of a previous systematic review, identify as such
Registration	2,4	2	If registered, provide the name of the registry (such as PROSPERO) and registration number
Authors:			
Contact	1	3a	Provide name, institutional affiliation, e-mail address of all protocol authors; provide physical mailing address of corresponding author
Contributions	9	3b	Describe contributions of protocol authors and identify the guarantor of the review
Amendments	NA	4	If the protocol represents an amendment of a previously completed or published protocol, identify as such and list changes; otherwise, state plan for documenting important protocol amendments
Support:			
Sources	9	5a	Indicate sources of financial or other support for the review
Sponsor	9	5b	Provide name for the review funder and/or sponsor
Role of sponsor or funder	9	5c	Describe roles of funder(s), sponsor(s), and/or institution(s), if any, in developing the protocol
**INTRODUCTION**			
Rationale	3	6	Describe the rationale for the review in the context of what is already known
Objectives	3	7	Provide an explicit statement of the question(s) the review will address with reference to participants, interventions, comparators, and outcomes (PICO)
**METHODS**			
Eligibility criteria	4	8	Specify the study characteristics (such as PICO, study design, setting, time frame) and report characteristics (such as years considered, language, publication status) to be used as criteria for eligibility for the review
Information sources	5 Online sup 2	9	Describe all intended information sources (such as electronic databases, contact with study authors, trial registers or other grey literature sources) with planned dates of coverage
Search strategy	5 Online sup 2	10	Present draft of search strategy to be used for at least one electronic database, including planned limits, such that it could be repeated
Study records:			
Data management	5	11a	Describe the mechanism(s) that will be used to manage records and data throughout the review
Selection process	5	11b	State the process that will be used for selecting studies (such as two independent reviewers) through each phase of the review (that is, screening, eligibility and inclusion in meta-analysis)
Data collection	5	11c	Describe planned method of extracting data from reports (such as

For peer review only

process			piloting forms, done independently, in duplicate), any processes for obtaining and confirming data from investigators
Data items	4, 5	12	List and define all variables for which data will be sought (such as PICO items, funding sources), any pre-planned data assumptions and simplifications
Outcomes and prioritization	4, 5	13	List and define all outcomes for which data will be sought, including prioritization of main and additional outcomes, with rationale
Risk of bias in individual studies	6	14	Describe anticipated methods for assessing risk of bias of individual studies, including whether this will be done at the outcome or study level, or both; state how this information will be used in data synthesis
Data synthesis	7	15a	Describe criteria under which study data will be quantitatively synthesised
	7	15b	If data are appropriate for quantitative synthesis, describe planned summary measures, methods of handling data and methods of combining data from studies, including any planned exploration of consistency (such as I^2, Kendall's τ)
	7, 8	15c	Describe any proposed additional analyses (such as sensitivity or subgroup analyses, meta-regression)
	7	15d	If quantitative synthesis is not appropriate, describe the type of summary planned
Meta-bias(es)	NA	16	Specify any planned assessment of meta-bias(es) (such as publication bias across studies, selective reporting within studies)
Confidence in cumulative evidence	6	17	Describe how the strength of the body of evidence will be assessed (such as GRADE)

For peer review only
